# Bio-Evaluation of the Wound Healing Activity of *Artemisia judaica* L. as Part of the Plant’s Use in Traditional Medicine; Phytochemical, Antioxidant, Anti-Inflammatory, and Antibiofilm Properties of the Plant’s Essential Oils

**DOI:** 10.3390/antiox11020332

**Published:** 2022-02-08

**Authors:** Hamdoon A. Mohammed, Kamal A. Qureshi, Hussein M. Ali, Mohsen S. Al-Omar, Omar Khan, Salman A. A. Mohammed

**Affiliations:** 1 Department of Medicinal Chemistry and Pharmacognosy, College of Pharmacy, Qassim University, Buraydah 51452, Saudi Arabia; m.omar@qu.edu.sa; 2 Department of Pharmacognosy, Faculty of Pharmacy, Al-Azhar University, Cairo 11371, Egypt; 3 Department of Pharmaceutics, Unaizah College of Pharmacy, Qassim University, Unaizah 51911, Saudi Arabia; ka.qurishe@qu.edu.sa; 4 Department of Pharmacology and Toxicology, College of Pharmacy, Qassim University, Buraydah 51452, Saudi Arabia; hu.ali@qu.edu.sa; 5 Department of Biochemistry, Faculty of Medicine, Al-Azhar University, Assiut 71524, Egypt; 6 Department of Medicinal Chemistry and Pharmacognosy, Faculty of Pharmacy, Jordan University of Science and Technology (JUST), Irbid 22110, Jordan; 7 Department of Pharmaceutics, College of Pharmacy, Qassim University, Buraydah 51452, Saudi Arabia; ok.mohamad@qu.edu.sa

**Keywords:** *Artemisia judaica*, essential oil, wound healing, inflammatory markers, antibiofilm activity, antioxidant activity

## Abstract

*Artemisia judaica* (ArJ) is a Mediterranean aromatic plant used traditionally to treat gastrointestinal ailments, skin diseases, atherosclerosis, and as an immuno-stimulant. This study describes ArJ essential oil constituents and investigates their wound healing activity. The in vitro antioxidant and antibiofilm activities of ArJ essential oil were investigated. The in vivo pro/anti-inflammatory and oxidative/antioxidant markers were compared with standard silver sulfadiazine (SS) in a second-degree skin burn experimental rat model. The gas chromatography-equipped flame ionization detector (GC-FID) analysis of ArJ essential oil revealed the major classes of compounds as oxygenated monoterpenes (>57%) and cinnamic acid derivatives (18.03%). The antimicrobial tests of ArJ essential oil revealed that *Bacillus cereus*, *Candida albicans*, and *Aspergillus niger* were the most susceptible test organisms. Two second-degree burns (each 1 inch square in diameter) were created on the dorsum of rats using an aluminum cylinder heated to 120 °C for 10 s. The wounds were treated either with ArJ or SS ointments for 21 days, while the negative control remained untreated, and biopsies were obtained for histological and biochemical analysis. The ArJ group demonstrated a significant increase in antioxidant superoxide dismutase (SOD) and catalase (CAT) enzymatic activities, while lipid peroxide (LP) levels remained insignificant compared to the negative control group. Additionally, ArJ and SS groups demonstrated a significant decrease in inflammatory levels of tumor necrosis factor α (TNF-α) compared to the negative group, while interleukin 1 beta (IL-1b) and IL-6 were comparable to the negative group. At the same time, anti-inflammatory IL-10 and transforming growth factor beta 1 (TGF-b1) markers increased significantly in the ArJ group compared to the negative control. The ArJ results demonstrated potent wound healing effects, comparable to SS, attributable to antioxidant and anti-inflammatory effects as well as a high proportion of oxygenated monoterpenes and cinnamate derivatives.

## 1. Introduction

*Artemisia* is a genus of annual, perennial, and biennial herbs in the Asteraceae (Compositae) family [1]. The plants of the genus *Artemisia* are frequently used in traditional medicine as remedies for human and animal ailments. For instance, *Artemisia* species have been used in traditional medicine for respiratory disorders, including coughs and phlegm, as a pain killer, worm expelling agent, diaphoretic and diuretic agent, and for the treatment of wounds, hypertension, and allergies [2]. In addition, some of the *Artemisia* plants are traditionally used to treat seizures, and the activity is confirmed through in vivo animal experiments [3,4,5]. *Artemisia* species have been reported in in vitro and in vivo experiments and in clinical trials evaluating their anticancer, antimalarial, antimicrobial, and antiviral activities [6,7].

Furthermore, several side effects and misuses have also been reported for some of the genus’ plants. For instance, *A. monosperma* leaves are not recommended in pregnancy and are used to induce abortion in Jordan [8]. However, this plant, in addition to other plants of *Artemisia*, e.g., *A. vulgaris*, has been used in folklore medicine for labor induction [8,9,10]. Besides abortion, vomiting, diarrhea, headache, pruritus, and rashes have been reported among young children and pregnant women who used *A. annua* to treat malaria [11]. The *Artemisia* plants’ biological activities were attributed to the presence of essential oils, sesquiterpene lactones, flavonoids, bitter principles, coumarins, and phenolic acids [1,2,12,13]. Several *Artemisia* species grow wildly or as cultivated plants for their use as medication and as a herbal tea preparation in the Mediterranean region [9,14,15].

*Artemisia judaica* L. (ArJ) is widely grown in the Mediterranean region, including Algeria, Libya, Egypt, Jordan, and Saudi Arabia [16,17,18,19,20]. In Saudi Arabia, ArJ grows in the kingdom’s northern region, including the border area of the Hail-Qassim regions [21]. ArJ has been reported for several traditional uses, e.g., healing external wounds and repairing snake and scorpion bites [22]. In addition, ArJ is traditionally used to treat gastrointestinal disorders, sexual inability, hyperglycemia, heart diseases, inflammatory disorders, arthritis, cancers [1,20], skin diseases, atherosclerosis, and enhance vision and immunity [23,24]. The Bedouins in Egypt (Sinai) and Saudi Arabia also use the plant as a herbal tea in treating GIT disorders [16]. Biologically, ArJ demonstrated antidiabetic, antioxidant, hepatoprotective, and anti-inflammatory activities in experimental animals [22,25,26] due to the properties inherent in the chemical structure of the compounds it contains [27]. The plant also exhibited weak antimicrobial activity against Gram-positive and Gram-negative bacteria [28,29]. In vitro studies reported the plant extract’s potential antioxidant and anticancer activities [28,29].

ArJ chemical analysis revealed the presence of flavonoids, e.g., glycosides and aglycones of apigenin, luteolin, and quercetin [22]. Other natural classes, such as phenolics, triterpenes, bitter principles, and sesquiterpene lactones, i.e., judaicin, have also been reported from the plant [22,30]. Additionally, ArJ is an aromatic plant. Its essential constituents have been identified from the plant species growing in different areas and climatic regions [18,20,23,24,31]; as well known as the anthropogenic factors, environmental conditions primarily affect the composition of the plant [32]. The overall analysis of the essential oil constituents of ArJ indicated that the monoterpene, i.e., piperitone, is the major chemotypic constituent in the plant from different genotypes [1,24]. In addition, other essential constituents of the plants, such as camphor, ethyl cinnamate, and spathulenol, have also been identified in relatively high concentrations in individual plant genotypes [24]. In addition, environmental conditions and the geographical locations of the plant growing areas have been reported to affect the major chemotypic constituents of ArJ essential oils. Table 1 demonstrates the major constituents of the plant essential oils from different locations.

The methods used for essential oil production from aromatic plants vary and mostly depend on the nature of the volatile constituents, the amount of the essential oils, and the nature of the plant samples [36]. Thereby, distillation procedures are primarily used for the plants containing a considerable amount of the thermostable volatile constituents; however, volatile (e.g., diethyl ether) and non-volatile (e.g., lard) solvent extraction processes are used for the extraction of the highly delicate aromatic plants which contain heat-sensitive and small quantities of the essential oils [37]. In addition, modern extraction techniques, such as CO_2,_ supercritical CO_2_ extraction and microwave-assisted extraction techniques, are used for the industrial-scale production of the essential oils with specific advantages, e.g., time- and quantity-based efficiency and environmentally friendly properties [38,39,40].

Burn injury traumas occur by friction, cold, heat, radiation, chemical, or electric sources, but hot liquids, solids, and fire contribute significantly towards burn injuries [41]. In Saudi Arabia, 52% of all burns occur in young children, and males are more prone to burns than females (1.42:1). Burn wounds require immediate attention to avoid hypovolemic shock and sepsis [42]. New approaches and drugs are being researched to facilitate faster burn wound healing [43], thereby minimizing adverse reactions, like allergy or irritation, due to topical agents that increase the rehabilitation period [44]. In addition to their general availability, herbal medicines have demonstrated a promising role in wound healing compared to silver sulfadiazine (SS) [45,46,47]. Nevertheless, modern approaches and methodologies are required to validate claims for herbal compounds [48].

The current study is designed to demonstrate the wound healing properties of ArJ essential oils as part of the plant’s use in traditional medicine. Therefore, a phytochemical analysis of the ArJ essential oil for the species growing in the Northern Qassim region of Saudi Arabia was conducted. The study also investigated the antioxidant, antimicrobial, and antibiofilm activities of the plant essential oil as associated analyses related to the wound healing potential of the plant.

## 2. Materials and Methods

### 2.1. Plant Materials and Distillation Procedure

The aerial plant parts were collected during March 2020, in the morning, from the Northern Qassim region of Saudi Arabia and identified as *Artemisia judaica* L. by the taxonomists in the Department of Plant Production and Protection, College of Agriculture, Qassim University (Buraydah, Saudi Arabia). A sample of the plant with the registered number #090 was kept at the herbarium of the College of Pharmacy, Qassim University (Buraydah, Saudi Arabia). The plant was dried in the shade at room temperature for ten days before the distillation process. The plant materials, 200 g, were reduced to coarse powder form, backed to a 2 L conical flask with a stopper, and thoroughly mixed with 700 mL of distilled water. The flask was connected to the Clevenger apparatus and fixed over the heating mantel. The flask contents were allowed to boil for a continuous 5 h. The distillate essential oil was collected over anhydrous sodium sulfate and stored in an opaque glass vial in a −20 °C freezer.

### 2.2. GC-FID Analysis of the Essential Oil

A gas chromatography (Perkin Elmer Auto System XL, Waltham, MA, USA) equipped flame ionization detector (GC-FID) was used to analyze the essential oil of ArJ. The chromatographic separation of the oil samples was achieved on a fused silica capillary column ZB5 (60 m × 0.32 mm i.d. × 0.25 µm film thickness). The oven temperature was maintained initially at 50 °C and programmed from 50 to 240 °C at a rate of 3 °C/min. ArJ essential oil sample was dissolved in analytical grade diethyl ether (2.9 mg of the oil in 100 µL of the solvent). Then, 1 µL of the mixture was injected with a 1/20 split ratio. The helium was used as the carrier gas at a 1.1 mL/min flow rate. The injector and detector temperatures were 220 and 250 °C, respectively.

### 2.3. Gas Chromatography–Mass Spectroscopy Analysis of the Volatile Oil

The GC–MS analysis of ArJ essential oil was conducted using an Agilent 8890 GC system attached to a PAL RTC 120 auto-sampler and equipped with a mass detector, Agilent 9977B GC/MSD mass spectrometer (Agilent technology, Santa Clara, CA, USA). An HP-5 capillary column (30 m, 250 µm i.d., 0.25 µm film thickness) was used to separate target molecules. The initial column temperature (50 °C for 2 min, isothermal) was programmed up to 220 °C at a rate of 5 °C/min, and then 10 °C/min up to 280 °C and kept constant at 280 °C for 10 min (isothermal). The injector temperature was 230 °C. Helium was used as a carrier gas at 1 mL/min flow rate. All the mass spectra were recorded using the following conditions. The run time was about 65 min. The transfer line was set at 280 °C, and the ionization source and the mass analyzer temperatures were set at 230 and 150 °C, respectively. Diluted samples (1% *v*/*v*) were injected with split mode (split ratio 1:15).

### 2.4. Identification of the Essential Oil Constituents

The constituents of the oil were identified based on the experimental retention index (RI) calculated with references to a series of standard *n*-alkenes series (C8–C40) and the retention indexes reported for the ArJ essential constituents besides the reported retention indexes obtained for the analysis of different essential oils under similar GC experimental conditions. In addition, the National Institute of Standards and Technology (NIST-11) and mass fragmentation patterns of the peaks were also used to identify the compounds. The relative percentages of the constituents were calculated from the area under the peak obtained from the GC-FID chromatogram.

### 2.5. Antioxidant Activity of ArJ Essential Oil

#### 2.5.1. Total Antioxidant Capacity (TAC)

The method described by Aroua et al. [49] was followed to conduct this experiment. In brief, sulfuric acid (0.6 M) and ammonium molybdate (4 mM) in sodium phosphate buffer (28 mM) were mixed to prepare the molybdate reagent. Then, 3.6 mL of the molybdate reagent was mixed with 0.4 mL of ArJ essential oil (containing 200 µg of the oil) in a stoppered glass test tube. The tube was vortexed and warmed for 30 min at 90 °C in a water bath. After cooling, the absorbance of the developed blue color was recorded at 695 nm using a spectrophotometer against a blank prepared essential oil. The TAC of ArJ essential oil was calculated equivalent to the Trolox using the standard calibration curve.

#### 2.5.2. DPPH (2,2-Diphenyl-1-Picrylhydrazyl) Scavenging Activity (DPPH-SA)

The method was conducted according to Shimada et al. [50]: 1 mL of the diluted ArJ essential oil (containing 200 µg of the oil in methanol) was mixed with 1 mL of DPPH (prepared by dissolving 6 mg of the DPPH in 50 mL of methanol). The mixture absorbance was measured at 517 nm after 30 min of standing at room temperature in a dark place. The DPPH-SA was calculated equivalent to Trolox from three independent measurements.

#### 2.5.3. Ferric Reducing Antioxidant Power (FRAP) Assay

Minor modifications to the method of Benzie and Strain [51] were carried out to measure the FRAP of ArJ essential oil. FRAP working reagent was freshly prepared by adding TPTZ (2,4,6-Tris(2-pyridyl)-s-triazine, 10 mM prepared in 40 mM HCl) to FeCl_3_·6H_2_O (20 mM) and acetate buffer (300 mM, pH 3.6) in a ratio 1:1:10. Then, 2 mL of the FRAP reagent was added to 0.1 mL of the ArJ essential oil (containing 200 µg the oil), the mixtures were incubated for 30 min at room temperature, and the absorbance was recorded at 593 nm. The procedure was conducted in triplicate, and the prepared FRAP–Trolox calibration curve was used to calculate the extract activity as mg Trolox equivalent per gram of the used plant’s dried extract.

#### 2.5.4. Metal Chelating Activity Assay (MCA)

The ArJ essential oil ability to chelate iron compared to the EDTA was evaluated using Zengin et al.’s method [52]. Briefly, a mixture of the ArJ essential oil (2 mL of ethanol containing 200 µg of the oil) and ferrous chloride (25 µL, 2 mM) was added to 100 µL of ferrozine to inchoate the color. The mixture’s absorbance was recorded at 562 nm against a blank (2 mL of the ArJ essential oil plus 200 µL of the ferrous chloride without ferrozine). The standard calibration curve of EDTA was prepared, and the chelating activity of the ArJ essential oil was calculated in equivalents of the EDTA.

### 2.6. Antimicrobial Activity of ArJ Essential Oil

#### 2.6.1. Preliminary Antimicrobial Activity

The preliminary antimicrobial activity of ArJ essential oil was determined by the disc diffusion method [53]. Modified Mueller–Hinton agar (MMHA) and potato dextrose agar (PDA) were used as test media. MMHA plates were prepared according to the protocol mentioned in the literature [54]. The sterile paper discs (6 mm in diameter) were impregnated with 20 μL of pure ArJ essential oil and then used to evaluate the antimicrobial potential of ArJ essential oil against the selected human pathogens. Levofloxacin (5 µg/disc) and clotrimazole (50 µg/disc) were used as antibacterial and antifungal control (C) drugs. Each test organism’s inoculum was prepared in sterile tryptic soy broth (TSB), and the turbidity of each suspension was adjusted equal to 0.5 MacFarland standard, which is equal to 1.5 × 10^8^ colony forming units (CFU/mL) for bacteria, 1 × 10^6^–5 × 10^6^ CFU/mL for yeast and 4 × 10^5^ to 5 × 10^6^ CFU/mL for mold. Following that, 100 µL suspensions of each adjusted inoculum were poured individually over the surface of the test agar plates and then uniformly spread using sterile swabs. The prepared discs of ArJ and control drugs were then put on the inoculated plates. The plates were incubated at 35 °C for 24 h for bacteria and 48 h for fungi. After incubation, the diameters of inhibitory zones were calculated on a millimeter (mm) scale. Each test was performed in triplicate. The results are expressed in mm ± standard deviation (SD).

#### 2.6.2. Minimum Inhibitory Concentration (MIC) and Minimum Biocidal Concentration (MBC)

MIC was determined by the resazurin-based micro-broth dilution method, while MBC was performed following the standard spot inoculation method [53,55]. The inocula of each test bacteria were prepared in TSB, following the Clinical and Laboratory Standards Institute (CLSI) guidelines (https://clsi.org/, accessed on 1st December 2021), where the OD_600_ value (0.08–0.12) was adjusted, resulting in ~1 × 10^8^ CFU/mL. Then, adjusted inocula were further diluted by 1:100 in TSB, resulting in ~1 × 10^6^ CFU/mL. In contrast, the inocula of test fungi were prepared in potato dextrose broth (PDB) following the CLSI guidelines, where the OD_600_ value (0.08–0.12) was adjusted, the resulting stock suspension contained 1 × 10^6^ to 5 × 10^6^ CFU/mL for yeast and 4 × 10^5^ to 5 × 10^6^ CFU/mL for mold. A working yeast suspension was prepared by a 1:100 dilution followed by a 1:20 dilution of the stock suspension with PDB, resulting in 5.0 × 10^2^ to 2.5 × 10^3^ cells/mL, while a working mold suspension was prepared by a 1:50 dilution of the stock suspension with PDB, resulting in 0.8 × 10^4^ to 1 × 10^5^ cells/mL. The initial stock solution of ArJ essential oil was prepared in DMSO (dimethyl sulfoxide) at a 200 µL/mL concentration. Each well in column 1 was dispensed with 200 µL of stock solution of ArJ essential oil. At the same time, each well of columns 2 to 10 contained 100 µL of tryptic soy broth (TSB) for antibacterial evaluation, while for antifungal assessment, 100 µL of potato dextrose broth (PDB) was used. A two-fold serial dilution of ArJ essential oil was made from columns 1 to 10 using a multichannel micropipette, resulting in concentrations of ArJ essential oil ranging from 200–0.39 µL/mL in columns 1 to 10. Column 11 had 200 µL of standardized inoculum suspensions, which served as negative control (NC), and column 12 had 200 µL of sterile broth, which served as sterility control (SC). Each organism’s adjusted inoculum was dispensed, 100 µL into each test well in columns 1–10, respectively. The 100 µL of adjusted microbial inocula were dispensed in all the wells of columns 1 to 10, resulting in ~5 × 10^5^ CFU/mL for bacteria and ~2.5 × 10^2^ to 1.25 × 10^3^ CFU/mL for *C. albicans*, and 0.4 × 10^4^ to 5 × 10^4^ CFU/mL for *A. niger*. At this stage, the final concentrations of ArJ essential oil were 100 to 0.195 µL/mL in columns 1 to 10. The time taken to prepare and dispense the OD-adjusted microbial inocula did not exceed 15 min. The inoculated plates were incubated at 35 °C for 24 h for bacteria and 48 h for fungi. Following incubation, 30 µL of sterile resazurin dye (0.015% *w*/*v*) was dispensed into each well of columns 1 to 12, and then plates were re-incubated for 1–2 h to observe color change. After incubation, columns with the lowest concentrations showing no color change (blue resazurin color stayed intact) were scored as MIC.

MBC was determined by directly plating the contents of wells with concentrations above the MIC on sterile tryptic soy agar (TSA) plates for bacteria, while potato dextrose agar (PDA) plates were used for fungi. The contents from the wells, which did not change from blue to pink, were inoculated on sterile tryptic soy agar (TSA) plates and incubated at 35 °C for 24 h for bacteria and 48 h for fungi. The lowest concentration of ArJ did not produce isolated colonies of the test organisms on inoculated agar plates considered as the MBC. The results are recorded in µL/mL.

#### 2.6.3. Minimum Biofilm Inhibitory Concentration (MBIC) and Minimum Biofilm Eradication Concentration (MBEC)

##### MBIC Assay

MBIC is defined as the lowest concentration of the antimicrobial agent (ArJ), preventing the biofilm formation of the tested organism. MBIC was conducted against the bacteria only. The 96-well microtiter plate was used to evaluate the anti-biofilm activity of ArJ [54]. The inocula of the test organisms were prepared in TSB equal to 0.5 MacFarland standard (1–2 × 10^8^ CFU/mL). An aliquot of 100 µL from the adjusted inocula was dispensed into each test well of a 96-well plate. Then 100 µL of different concentrations of ArJ were dispensed into test wells. Thus, the final concentrations for MBIC assessment were MIC, 2 × MIC, and 4 × MIC. The wells containing only 200 µL of TSB served as a blank control (BC), whereas those containing bacterial cultures without ArJ served as negative control (NC). The plates were incubated in a shaking water bath at 35 °C for 24 h at 100 rpm shaking speed. After incubation, the supernatants from each well were decanted gently by reversing the plates on a tissue paper bed/or removed by a pipette without disturbing the biofilms. The plates were dried in air for 30 min, stained with 0.1% (*w*/*v*) crystal violet at room temperature for 30 min, and then washed three times with distilled water. Subsequently, the crystal violet was solubilized by adding 200 µL of 95% ethanol to each test well. The absorbance was recorded in a microplate reader (xMark™ Microplate Absorbance Spectrophotometer-Bio-Rad, Hercules, CA, USA) at 650 nm. The lowest concentration of ArJ at which the absorbance equals or falls below the negative control is considered MBIC. Each test was performed in triplicate. The mean of three independent tests was taken. The results are expressed in µL/mL.

##### MBEC Assay

MBEC is defined as the minimum concentration of an antimicrobial agent (ArJ) that eradicates the biofilm of the test organism [54]. A 200 µL (1–2 × 10^8^ CFU/mL) inoculum of each test organism was inoculated into each test well of a flat-bottom 96-well microtiter plate. The plates were incubated at 35 °C for 48 h in a shaking water bath at 100 rpm shaking speed for biofilm formation. After the biofilms had formed, the contents of the test wells were decanted gently by reversing the plates on a tissue paper bed/or removed by a pipette without disturbing the biofilms. The various concentrations, i.e., MIC, 2 × MIC, and 4 × MIC of ArJ, were added to different test wells (200 µL/well). The inoculated plates were re-incubated at 35 °C for 24 h. After incubation, the contents of each test well were discarded by inverting the plates on a tissue bed. The plates were dried in air for 30 min, and then 200 µL of sterile TSB was dispensed in each test well. Then, 30 µL of 0.015% *w/v* resazurin dye was added into each test well. The plates were re-incubated for 1–2 h. After re-incubation, the MBEC was recorded by observing the color change from blue to pink. The column with no color change (blue resazurin color stayed intact) was scored MBEC. Biofilm without ArJ served as a negative control (NC). Each test was performed in triplicate. The mean of three independent tests was taken. The results are expressed in µL/mL.

### 2.7. Preparation of Ointment Formulation Loaded ArJ Essential Oil

Ointment formulation of 5% *w*/*w* strength of ArJ essential oil was prepared. The simple ointment base was prepared by the fusion method according to the *British Pharmacopoeia 1988* [56]. Briefly, 100 g of simple ointment base was prepared by melting hard paraffin (5 g) in a beaker at 61 °C. The other ingredients, i.e., cetostearyl alcohol (5 g), wool fat (5 g), and soft white paraffin (85 g), were added in descending order of melting point. The homogenous mixture was removed from the heat and stirred until cold. Then, 5% *w*/*w* strength ArJ essential oil ointment was prepared by incorporating 5 g of the essential oil into 95 g of a simple ointment base in small portions by mixing with trituration using an ointment mortar and pestle. Finally, the ArJ ointment was transferred to a clean container. The control ointment, 50 g of the entire base ingredients, was taken and treated in the same way to formulate without the essential oil. The prepared ArJ ointment was physically examined and was consistent, homogenous, and stable for the measured one month.

### 2.8. In Vivo Wound Healing Animal Experiment

Twenty healthy 3-month-old Sprague Dawley female rats weighing about 150 ± 50 g were individually maintained in the cage under 25 ± 2°, 65% humidity, 12:12 light/dark cycle. Animals were fed with a standard chow diet with water ad libitum, and the wound healing study was conducted following the guidelines of the Institutional Animal Ethics Committee (Registration # 21-04-06). The animal groups involved intact, negative control, positive control (1% silver sulfadiazine, SS), and ArJ 5% ointment.

### 2.9. Skin Burn Induction Model

Briefly, the animals were anesthetized using xylazine 5 mg/kg and ketamine 50 mg/kg, and the rat’s dorsum was shaved with a hair trimmer (GEEPAS^®^, Guangzhou, China) at a 45° angle to minimize the angle skin injury during shaving and disinfected using 70% ethanol. An aluminum cylinder (1-inch square diameter, 86 g weight) was heated using a hot water bath at 120 °C for at least 60 min to ensure thermal equilibrium with the water. The exact temperature of the cylinder and the water was measured before inducing the burns using a dual probe thermometer (UT320D Mini Contact Type Thermometer Dual Channel K/J Thermocouple, UNI-T, Dongguan, China). Second-degree burns of 1-inch square diameter were induced on the rat’s shaved dorsum by patching the aluminum cylinder on the rat’s dorsum for 10 s, allowing it to stand on its own weight to ensure symmetrical burns across all rats [57,58,59]. The animals were administered with 0.9% normal saline i.p injection 10 mL/kg. The treated groups were applied topically twice daily for three weeks with ArJ 5% ointment or 1% SS cream topically on the wound area.

### 2.10. Biopsy

At the end of the experiment on day 21, the animals were euthanized and a biopsy measuring 1 × 1 cm diameter was collected using scissors and tweezers from the underlying tissue. One part of the biopsy was fixed in 3.7% formalin for paraffin embedding, while another part was homogenized and the supernatant isolated and stored at −20 °C for biochemistry analysis.

### 2.11. Histological Staining

Tissue was processed within 48 h of collection by dehydration with increasing ethanol percentages before being cleared with xylene and embedded in paraffin wax. Tissue sections of 5 microns were cut using a microtome (MEDIMEAS, Haryana, India), allowing simultaneous sectioning of the epidermis and dermis. Sections were stained using hematoxylin and eosin (H&E) and visualized under a light microscope at 40× magnification. Five fields/sections were counted for the amount of fibroblast, collagen, inflammation, and neovascularization, and the data was scored from 0–4, where 0, 1, 2, 3, and 4 represented normal, low, moderate, high, and very high, respectively, as described previously [60].

### 2.12. Determination of Oxidants and Antioxidants

The catalase (CAT, Serial No. 24IF07D5A0) and superoxide dismutase (SOD, Serial No. 745402C55B) activity, and lipid peroxide (LP, malondialdehyde, Serial No. 1F4346D808) levels were determined in skin wound tissue homogenate by enzyme-linked immunosorbent assay (ELISA) kits (Cloud Clone Corp Company, Houston, TX, USA), according to the manufacturer’s instructions. The absorbance was measured at 450 nm by a microplate ELISA reader and the concentration was calculated using a standard curve.

### 2.13. Determination of Pro-Inflammatory and Anti-Inflammatory Cytokine Levels: Interleukins, TGF-b, and TNF-α Levels

The pro-inflammatory cytokines: interleukin 1 beta (IL-1b, Serial No. 282D397BBC), IL-6 (Serial No. 51D9580378), and TNF-α (Serial No. 3898289A45) and the anti-inflammatory cytokines (IL-10 (Serial No. 6AB644B25F), transforming growth factor beta 1 (TGF-b1, Serial No. 34997E20C3) were assayed in tissue homogenate by ELISA kits (Cloud Clone Corp Company, Houston, TX, USA) according to the manufacturer’s instructions. The microplates were measured with a 450 nm filter by a microplate reader.

### 2.14. Wound Area Measurement

Using a standard camera, images of skin burn for all the animals were captured on the day of burn induction and at different time points (week 1, 2, and 3), while the wound measurement was performed before the treatment and 2 weeks after the treatment using freely available Image J software (version 1.8.1, Public Domain, Madison, WI, USA). Due to hair regrowth on the wound area, wound size could not be measured accurately after 2 weeks.

### 2.15. Statistical Analysis

Data were expressed as the mean ± standard error of the mean (SEM) (*n* = 5). Differences between groups were analyzed using one-way ANOVA, except for wound area measurement at different time-points, which was analyzed using two-way ANOVA followed by a post hoc test using Tukey’s multi-group comparison on GraphPad Prism 8.0.2 (GraphPad Software, San Diego, CA, USA). The data were considered significant if *p* < 0.05 [61]. The superscripts (A–C) describing significance among the groups in the tables were obtained using Minitab 19.1 (Minitab LLC, State College, PA, USA).

## 3. Results and Discussion

### 3.1. Essential Oil Constituents of A. judaica

Several parameters have been reported as influencing factors affecting essential oil production, constituents, and quality; the parameters include the maturity stage of the plant, the oil extraction processes, and the drying methods applied to the aromatic plant samples, as well as the environmental conditions where the aromatic plant grows [62,63,64,65]. The essential oil of wild ArJ growing in the Northern Qassim region of Saudi Arabia has been isolated by the hydro-distillation technique using a Clevenger apparatus from the shade-dried aerial parts of the plant. Three different distillation experiments have been used to calculate the essential oil production percentage of 1.71 ± 0.3% *w*/*w* of the dried plant aerial parts. The percentage yield was higher than the reported yields for the cultivated species of the plant growing in Saudi Arabia (0.18% *v*/*w*) [24], indicating the higher capacity of the wild species of ArJ to biosynthesize essential oils. In addition, the nature of the plant sample, i.e., fresh or dried, and the conditions of the drying process could be factors affecting oil production percentage. The reported oil production percentage (0.18% *v*/*w*) has been calculated for the fresh plant samples [24]. However, the current percentage (1.71 ± 0.3% *w*/*w*) of essential oil production resulted from the distillation of the ten-day dried plant sample, which is consistent with the reported percentages of the essential oil production from dried samples of the aromatic plants, i.e., rosemary and sage [66,67]. Moreover, the current essential oil recovery percentage (1.71 ± 0.3% *w*/*w*) was nearly similar to the recorded data reported for the wild species of ArJ growing in the Southern region of Jordan (1.62%) [20].

**Figure 1:**
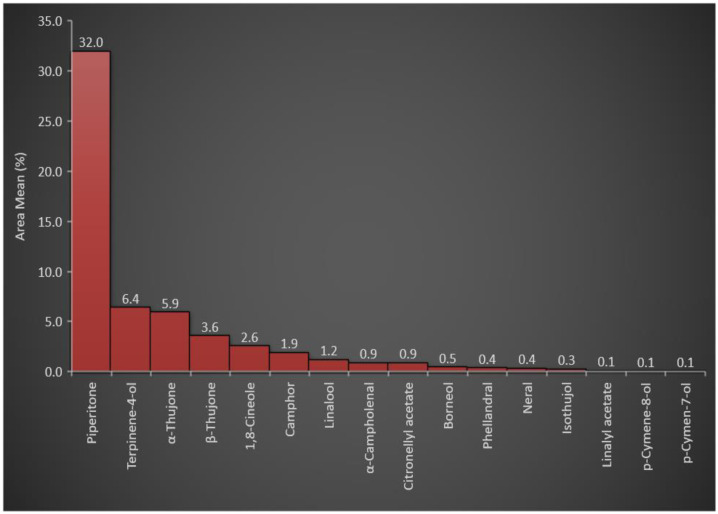
Representation of the oxygenated monoterpenes in *A. judaica* essential oil.

The produced oil samples obtained from each distillation experiment were independently subjected to GC-FID analysis (Supplementary file, Figure S1). The results expressed in Table 1 show the mean relative percentage of the individual compounds plus standard deviations obtained from the three GC-FID spectroscopic runs. Kovats retention index was calculated with the C_8_–C_40_ series of *n*-alkenes analyzed under identical extermination conditions. The reported retention indexes were also used to identify the ArJ essential constituents. The results shown in Table 2 indicated that oxygenated monoterpenes represented ≈ 57% of the plants’ essential constituents among all essential oil classes. The higher percentage of oxygenated monoterpenes was attributed to the presence of piperitone in a high concentration (31.99% of the total essential oils in the plant). In addition, other oxygenated monoterpenes, e.g., terpinene-4-ol, α-thujone, β-thujone, 1,8-cineole, camphor, and linalool, were represented at relatively high concentrations of 6.42, 5.94, 3.61, 2.56, 1.92, and 1.21%, respectively, with a total percentage of 21.66%. The concentration of piperitone (31.99%) and the total oxygenated monoterpene concentrations (57%) among the total essential oil constituents (Figure 1) were consistent with the reported chemotypic properties of the plant [24] that have been found, 30–70% of piperitone in the essential oil of ArJ growing wildly in different regions of the Mediterranean countries, such as Egypt, Algeria, and Jordan [20,31,68,69].

Besides the monoterpenes, GC-FID analysis of ArJ also showed a comparatively high percentage of cinnamic acid derivatives (18.03%), represented by the presence of three essential constituents, i.e., (*E*)-methyl cinnamate (0.35%), *cis*-ethyl cinnamate (4.02%), and *trans*-ethyl cinnamate (13.67%). Notably, ethyl cinnamate has been reported as one of the major chemotypes of the plant [24]. Monoterpene hydrocarbons, sesquiterpene hydrocarbons, oxygenated sesquiterpenes, and phenolic essential oils were also represented in the essential oil to a lesser extent, with 5.74, 10.88, 4.66, and 1.87%, respectively (Table 2).

### 3.2. In Vitro Antioxidant Activity of the ArJ Essential Oil

Antioxidants are promising therapeutic agents in wound healing [70]. Most of the reported plants with wound healing activity possess noticeable antioxidant potency, which has been examined by different in vitro and in vivo assays [71]. The essential oils obtained from several plants of the genus *Artemisia*, e.g., *A. diffusa* and *A. herba-alba*, have exhibited potential free radical scavenging, reducing, and metal-chelating properties [72,73]. The measurements, i.e., TAC, DPPH-SA, FRAP, and MCA, were conducted for the essential oil of ArJ quantitatively. The plant’s essential oil reduced the molybdate ions (VI) to molybdenum (V) in the TAC assay at a level of 59.32 mg of Trolox equivalents per gram of the plant essential oil.

Moreover, the ArJ essential oil exhibited notable reducing characteristics towards the ferric ion measured by the FRAP assay (22.34 mg of Trolox equivalent per gram of the essential oil). This ArJ essential oil-reducing characteristic in the TAC and FRAP contributes to the overall activity of this oil as an antioxidant agent [74]. The noticeable reducing characteristic of the ArJ essential oil could be attributed to the presence of camphor (1.92%), ethyl cinnamate (4.02%), and piperitone (31.99%) in relatively high concentrations [75,76,77]. The results also revealed that the essential oil of ArJ can chelate iron by 26.99 mg of EDTA equivalents per gram of essential oil, which was consistent with the reported ferrous ion-chelating activity of ArJ [35]. As iron has a primary role in the Fenton reaction involving the conversion of the oxidizing agent hydrogen peroxide (H2O2) into the more reactive hydroxyl radical (HO·), the iron-chelating agents, such as the ArJ essential oil, interfere with the progression and exaggeration of the oxidative stress [78].

Furthermore, scavenging activity of ArJ essential oil has been reported [35,69]. In the current study, the essential oil of the ArJ also exhibited scavenging activity, measured as 10.70 mg of Trolox equivalent per gram of the essential oil against the stable free radical DPPH. The results of the antioxidant activity of the plant essential oil seems also to be attributable to the presence of considerable percentages of oxygenated monoterpenes, cinnamate derivatives, and phenolics in the essential oils of the plants, all of which are known for their antioxidant activity [79,80,81]. The overall results obtained from quantitative in vitro antioxidant assays confirmed the antioxidant activity of the ArJ essential oil and supported the association between the wound healing potential of the plant and its antioxidant activity.

### 3.3. Antimicrobial Profile of ArJ Essential Oil

#### 3.3.1. Preliminary Antimicrobial Activity

**Figure 2:**
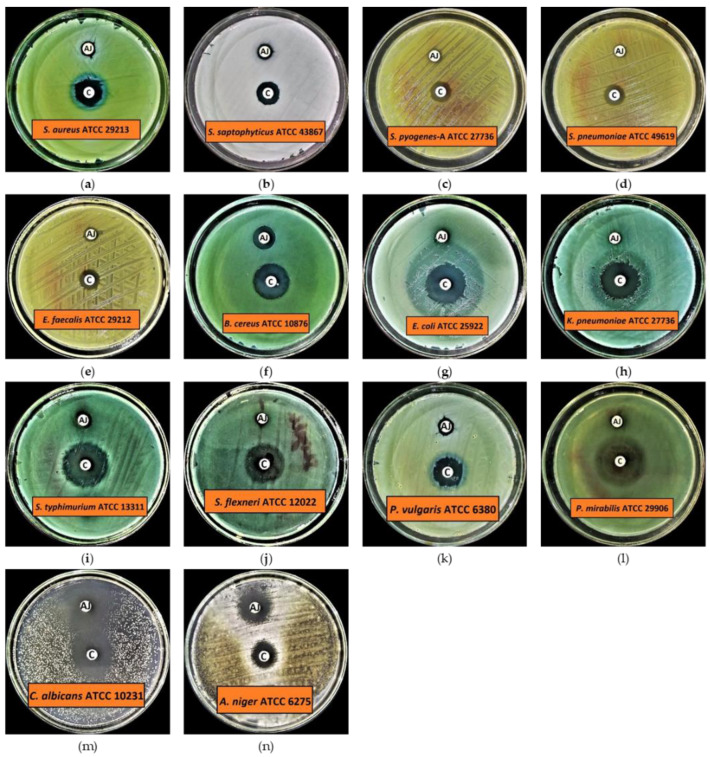
Preliminary antimicrobial activity of ArJ essential oil. (**a**) *Staphylococcus aureus* (*S. aureus*) ATCC 29213; (**b**) *Staphylococcus saptophyticus* (*S. saptophyticus*) ATCC 43867; (**c**) *Streptococcus pyogenes* (*S. pyogenes*)-A ATCC 27736; (**d**) *Streptococcus pneumoniae* (*S. pneumoniae*) ATCC 49619; (**e**) *Enterococcus faecalis* (*E. faecalis*) ATCC 29212; (**f**) *Bacillus cereus* (*B. cereus*) ATCC 10876; (**g**) *Escherichia coli* (*E. coli*) ATCC 25922; (**h**) *Klebsiella pneumonie* (*K. pneumoniae*) ATCC 27736; (**i**) *Salmonella typhimurium* (*S. typhimurium*) ATCC 13311; (**j**) *Shigella flexneri* (*S. flexneri*) ATCC 12022; (**k**) *Proteus vulgaris* (*P. vulgaris*) ATCC 6380; (**l**) *Proteus mirabilis* (*P. mirabilis*) ATCC 29906; (**m**) *Candida albicans* (*C. albicans*) ATCC 10231; (**n**) *Aspergillus niger* (*A. niger*) ATCC 6275. AJ referred to Artemisia judaica essential oil, while C referred to the drug control.

The results of preliminary antibacterial activity demonstrated that all the tested organisms, including Gram-positive and Gram-negative bacteria, are susceptible to ArJ essential oil, except *Pseudomonas aerugenosa* ATCC 9027, which showed resistance at the given concentration of ArJ essential oil, i.e., 20 μL/disc (Figure 2 and Table 3). The results further demonstrated that *Bacillus cereus* is a highly susceptible test organism, with an inhibition zone of 12.9 ± 0.10 mm at the given concentration of ArJ essential oil. In contrast, the lowest antibacterial activity was observed against *Klebsiella pneumoniae* and *Shigella flexneri*, with inhibition zones of 6.2 ± 0.10 mm and 6.2 ± 0.10 mm in diameter, respectively (Figure 2 and Table 3). Additionally, the findings indicated that the range for the mean zone of inhibition for Gram-positive bacteria is 7.2–12.9 mm, while for Gram-negative bacteria it is 6.2–10.0 mm, indicating that Gram-positive bacteria are more susceptible than Gram-negative bacteria to a given dose of ArJ essential oil.

The results for preliminary antifungal activity indicate that both the tested fungal strains are highly susceptible to ArJ essential oil. The results also indicated that the highest antifungal activity was observed against *Candida albicans* with an inhibition zone of 25.2 ± 0.20 mm, while *Aspergillus niger* had an inhibition zone of 15.0 ± 0.20 mm at the given concentration of ArJ essential oil. The control antibiotics inhibited the growth of all the tested organisms at the given concentrations, i.e., 5 μg/disc for levofloxacin and 50 μg/disc for clotrimazole, respectively (Figure 2 and Table 3).

#### 3.3.2. Minimum Inhibitory Concentration (MIC), Minimum Biocidal Concentration (MBC), Minimum Biofilm Inhibitory Concentration (MBIC), and Minimum Biofilm Eradication Concentration (MBEC)

The MIC and MBC results for the tested bacteria revealed that the MIC values ranged from 6.25 to 100 µL/mL, while MBC values ranged from 12.5 to >100 µL/mL (Table 4). The MIC and MBC results for the tested fungi demonstrated that *Candida albicans* had MIC and MBC values of 3.125 µL/mL and 6.25 µL/mL, respectively, whereas *Aspergillus niger* had values of 6.25 µL/mL and 12.5 µL/mL, respectively. The MBIC and MBEC results revealed that the MBIC values for the tested bacteria ranged from 6.25 to 100 µL/mL, whereas the MBEC values ranged from 12.5 to 200 µL/mL (Table 4).

Our findings for ArJ essential oil antimicrobial activity are consistent with previously published data [17,24,82,83,84,85]. Benmansour et al. demonstrated that ArJ essential oil had an excellent inhibitory effect against tested MRSA (methicillin-resistant *Staphylococcus aureus*), *S. aureus*, and *B. subtilis* [17], which is consistent with our results. Benderradji et al. showed that petroleum ether and ethyl acetate extracts of *A. sahariensis* leaves had the highest inhibitory activity against most tested strains. The most reported significant inhibition zone was obtained with chloroform extract of the plant against *Pseudomonas* [82]. These findings partially corroborate our results, since ArJ essential oil could not kill *Pseudomonas*, which might be a consequence of the essential oil and extract’s differing phytochemical contents, as well as species variations. Elazzouzia et al. demonstrated that the essential oil of *A. ifranensis* had highly potent antibacterial activity against the tested *S. aureus* [55], which is, again, consistent with our results. Kazemi et al. demonstrated that the essential oil of the aerial parts of *A. kermanensis* had highly potent antibacterial activity against *B. subtilis*, *P. aeruginosa*, and *S. aureus*, which is partially consistent with our results [85]. Al-Wahaibi et al. demonstrated that essential oils derived from *A. judaica* and *A. herba-alba* had potent antimicrobial potential against the tested organisms, including *Aspergillus fumigatus, Syncephalastrum racemosum, Geotricum candidum Candida albicans, Streptococcus pneumoniae, Bacillus subtilis,* and *Escherichia coli,* except *Pseudomonas aeruginosa*; these results are consistent with our results [24]. The results of our study indicated that ArJ essential oil has highly potent antimicrobial activity, demonstrating that ArJ essential oil could be a promising antimicrobial drug candidate and can cure various human infections, e.g., wound infections, boils, acne, etc., caused by various life-threatening pathogens, including bacteria and fungi. These results encouraged us to conduct wound healing testing on an animal model to verify the antimicrobial properties of ArJ essential oil in-vivo.

### 3.4. In Vivo Skin Burn Wound Healing

In the current study, the second-degree burn was induced on female rats based on the recent publication that observed significant wound healing in second and third-degree wounds [57]. The choice of three-month-old female rats was due to their quicker wound healing and greater wound contraction ability as compared to males [86].

#### 3.4.1. Morphological Appearance and Histological Analysis of the Wounds

**Figure 3:**
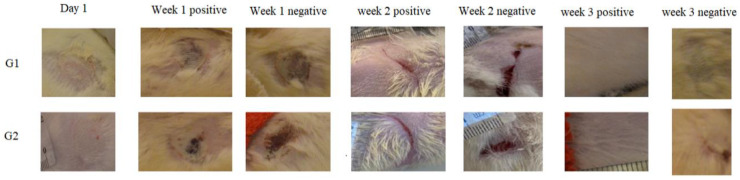
Morphological appearances of *Artemisia judaica* (G1)- and silver sulfadiazine (SS) (G2)-treated wounds at various time points.

The observations of wounds over three weeks of treatment revealed the significant progression in the healing process among the treated groups. At the time of burn induction, the skin burns produced were whitish in color and round in shape. After one week of the treatment, a crust developed on the wound along with the disappearance of edema in the treated groups (ArJ and SS groups). The wound area started decreasing by the second week of the treatment; however, edema formation was still prominent in the untreated burn area. At the end of 3 weeks, the treatment wound area for both the ArJ and SS groups demonstrated recovery, while the untreated zone did not recover completely (Figure 3).

Wound areas were measured using freely available Image J software. No significant differences were observed among the groups after the induction of skin burn. Two weeks after the treatment, the ArJ group’s wound area decreased significantly (*p* = 0.04), while the SS group’s wound size remained insignificant compared to the negative group (Supplementary file, Figure S2).

Wound healing is a complex restorative process of injured tissue to its original state [87], and it involves hemostasis, inflammation, proliferation, and remodeling [88] to prevent complex metabolic alteration affecting body organ systems. During the process of hemostasis, blood coagulation occurs, while the inflammation process ensures safety from invasive pathogens [88], thereby facilitating the proliferation step [89] towards remodeling the tissue maturation process [89,90]. During skin burn, cells and tissues are damaged substantially, thereby involving a complicated healing network compared to wound incision [87]. Based on the deepness of the burn wounds, they are categorized as first-, second-, and third-degree burns. The first-degree burn is generally red or gray without any blisters and normal capillary network, while in a second-degree burn, blisters and partial-thickness damage to the dermis are observed. Second- and third-degree burns are treated similarly [91]. In the current study, second-degree skin burn wounds were induced, which healed over 3 weeks for the ArJ and SS groups.

**Figure 4:**
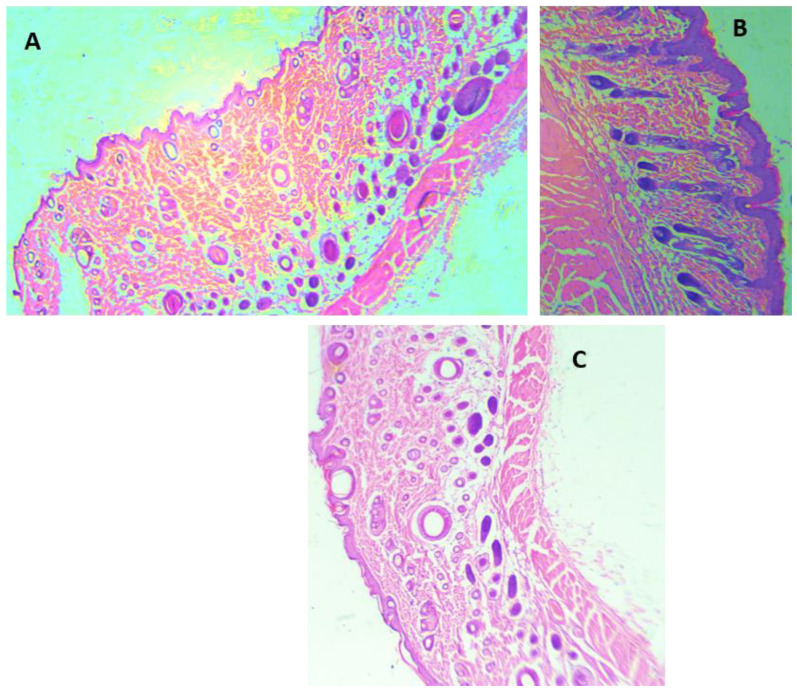
Histological analysis using hematoxylin and eosin staining of control (**A**), *Artemisia judaica* (**B**), and SS (**C**). Arrows (black) indicate neutrophil infiltration; magnification 40×.

H&E staining was performed for all the animal groups (Figure 4). The H&E staining demonstrated epidermis integrity and the degree of neutrophilic infiltration in the dermis and capillaries of the ArJ and SS groups compared to the untreated wound zone. Wound healing visual appearance for ArJ was not enlarged, most probably due to the balm effect of paraffin (Figure 4B). Tissue sections were analyzed qualitatively for the amount of fibroblast, collagen, inflammation, and neovascularization in SS and ArJ groups. The data demonstrated increased collagen, fibroblast, and neovascularization, with decreased inflammation in the SS and ArJ groups compared with the negative control group (Supplementary file, Figure S3).

#### 3.4.2. Role of Antioxidants and Oxidative Stress Markers in Wound Healing

The ArJ ointment group demonstrated significantly increased antioxidant SOD (*p* = 0.03) and CAT (*p* < 0.01) enzymatic activities compared to the negative group. The SOD and CAT activities were comparable in the intact and negative control. The SS treated group demonstrated a significant difference in CAT activity compared to the negative group (*p* = 0.01) and was comparable to the ArJ group, while the differences were insignificant for SOD activity. The antioxidant activity contributing towards wound healing is in accordance with previous studies reporting enhanced wound healing due to potent antioxidant activities [92]. LP significantly increased in the negative control group (*p* < 0.0001) compared to the intact group, which accords with previously recorded data [93]. No significant differences in LP were observed in treatment groups with either ArJ or SS groups compared to the negative control, while the ArJ and SS treated groups exhibited significant increases (*p* < 0.0001) compared to the intact group (Table 5).

#### 3.4.3. Role of Pro- and Anti-Inflammatory Markers in Wound Healing

The pro-inflammatory markers IL-1b and IL-6 values were comparable among all the studied groups, similar to the previously published articles [94,95]. At the same time, tumor necrosis factor α (TNF-α) significantly increased in the negative control group compared to the intact group (*p* < 0.0001). TNF-α values decreased significantly after treatment with SS (*p* < 0.02) and ArJ (*p* < 0.002) compared to the negative control group. TNF-α, the inflammatory cytokine activated during acute inflammation by macrophages/monocytes, plays a vital role in cell signaling, leading to necrosis or apoptosis. TNF-α participates in vasodilatation and edema formation and leukocyte adhesion to the epithelium through the expression of adhesion molecules. Furthermore, TNF-α regulates blood coagulation, contributes to oxidative stress at sites of inflammation, and indirectly induces fever. The data conforms with Gushiken and Periera, where TNF-α decreased after two weeks of treatment in skin wound tissue compared to the negative control [94,95].

The anti-inflammatory or the pro-angiogenic markers IL-10 and transforming growth factor beta 1 (TGF-b1) increased significantly in both ArJ (*p* < 0.0001 and *p* < 0.0001) and SS (*p* < 0.0001 and *p* < 0.0001) groups compared to the negative and intact control groups. However, differences in IL-10 and TGF-b1 levels in the negative control group were insignificant compared to the intact group (Table 6). The data confirm previous studies where IL-10 increased significantly in skin wound healing tissue after two weeks of treatment compared to the negative control group [94,95].

Various plant and herbal products are economically cheap to procure and demonstrate modest therapeutic potency with minimum toxicity relative to synthetic drugs [45,46,47,96]. Eupolin ointment, derived from an aqueous extract of *C*. *odorata* leaves, is the first Vietnam FDA-approved product [59]. The wound healing mechanism, even though it remains unclear, nevertheless enhanced blood flow, decreased inflammatory response, and reduced infection rates, all of which are contributing factors to angiogenesis. Rats are loose-skinned, in contrast to the tight human skin, with quicker constriction of the wound than the epithelization process; as such, rat wound healing, even though it is resemblant, is not entirely similar to wound healing in human skin [97,98]. However, rats are widely used animal models due to their genetic and behavioral similarity, ease of handling, and being economically viable. Thus, the rat skin burn model serves as a vital knowledge resource.

Mortality has been one of the major concerns in patients with deep burns due to infections, and researchers have attempted to minimize wound infection risk and accelerate the healing process [58]. Topical antimicrobial ointments are commonly employed for such purposes as SS 1% with low toxicity and have a potent antibacterial effect in burn wound therapy management [99,100,101].

Increased antioxidant levels have demonstrated wound healing potency [102] by protecting tissue from oxidative stress [103,104]. In the current study, ArJ demonstrated antioxidant (augmented SOD and CAT) enzymatic activities, validating the role of antioxidant enzymes, in addition to potentiating wound healing through increased anti-inflammatory levels. TGF-b1 is involved in wound healing, angiogenesis, immune regulation, and cancer. On the contrary, TGF-b1, along with inflammatory marker IL-6, helps in T helper 17 differentiation (Th17), aggravating inflammation [105]. In our study, the levels of TGF-b1 increased significantly while no statistical difference was observed for IL-1b and IL-6 in the ArJ group compared to the control group.

Moreover, IL-10 is a key regulator of the immune system by limiting the inflammatory response, which could otherwise cause tissue damage. In one study, IL-10 knockout mice were found prone to colitis [106,107], and blocking of IL-10 resulted in severe pathology. On the contrary, increased IL-10 levels cause chronic infection, and IL-10 blocking paved the way for pathogen clearance [108]. Mucosal secretion of IL-10 and TNF-α were augmented during wound healing, demonstrating the protective effect of IL-10 against inflammation. On the contrary, treatment with *P. pinnata* increased the serum IL-10 concentration while downregulating TNF-α and IL-6 [45]. Our study showed a significant increase in IL-10 concomitant with a significant decrease in TNF-α in ArJ compared to the negative control group, thus confirming the potential role of IL-10 in promoting wound healing.

The current findings for the in vivo and in vitro antioxidant activity and the anti-inflammatory effect of ArJ could be attributed to the presence of higher percentages of oxygenated monoterpenes (57.2%) in the plant essential oils [109]. Oxygenated monoterpenes have been found as major constituents in the plants used traditionally to accelerate wound healing, e.g., the plant essential oil of *Helichrysum italicum* [110,111] has exhibited a primary role in potential antimicrobial activity and anti-inflammatory effects [111]. Furthermore, some of the major ArJ essential oils reported as antioxidant and anti-inflammatory agents, e.g., thujone (both α and β-thujone, 9.55%), 1,8-cineole (2.56%), camphor (1.92%), and borneol (0.47%), have chiefly contributed to the rosemary anti-inflammatory effect [112]. Furthermore, 1,8-cineole antioxidant and anti-inflammatory effects have been reported, and the compound effect as an inhibitor for the inflammatory markers, TNF-α, IL-6, IL-8, LTB 4, PGE 2, and IL-1β, as well as down-regulation of 5-lipoxygenase (LOX) and cyclooxygenase (COX) pathways, are well documented [113,114]. Moreover, methyl cinnamate, a major constituent in ArJ essential oil (4.02%), has demonstrated potent anti-inflammatory activity [115]. All these compounds participated in the antioxidant and anti-inflammatory effects of the ArJ essential oil as well as in the wound healing activity. However, other identified constituents in this article could also be playing a role in the demonstrated plant activities.

## 4. Conclusions

The phytochemical analysis of the ArJ essential oils was conducted and revealed the dominance of the highly active antioxidant volatile compounds, oxygenated monoterpenes, and cinnamic acid derivatives in the essential oil constituents of the plant. Such classes of compounds were reflected in the in vitro and in vivo potential antioxidant activity of the ArJ essential oil. In the current study, wound treatment with ArJ also demonstrated significantly increased SOD and CAT enzymatic activities, with insignificant LP levels compared to the negative control group. In addition, ArJ reduced the pro-inflammatory marker TNF-α and augmented pro-angiogenic/anti-inflammatory TGF-b1 and IL-10 levels. The antimicrobial and antibiofilm potential of ArJ essential oil against *Bacillus cereus, Candida albicans,* and *Aspergillus niger* confirmed in the study supports the effectiveness of ArJ essential oil as a wound healing candidate. These results validate the curative role of ArJ in the treatment of skin wounds, which is attributed to its antioxidant and anti-inflammatory effects, as well as its high proportion of oxygenated monoterpenes and cinnamate derivatives.

## Figures and Tables

**Table 1:** Major constituents of the ArJ essential oils from plant species growing in different areas.

Locations	Major Constituents	Y%	Ref.
Saudi Arabia	cis-Thujone (2.5%), thymol (3.5%), trans-sabinyl acetate (3.3%), carvacrol (3.5%), b-eudesmol (13.1%), eudesma-4 (15), 7-dien-1-b-ol (3.5%), and hexadecanoic acid (5.7%)	0.18% (*v*/*w*)	[24]
Algeria	Piperitone (66.17%), ethyl cinnamate isomer (6.11%), spathulenol (2.34%), E-longipinane (2.55%)	1.7% (*w*/*w*)	[33]
Egypt	Piperitone (49.1%) and camphor (34.5%), borneol (3.90%)		[34]
Sinai, Egypt	Camphor (31.4%), endo-borneol (5.72%), piperitone (29.9%)	0.28%	[18]
Jordan	Artemisia ketone (9–24%), chrysanthenone (4–31%), piperitone (3–15%), camphor (0.3–16%), cinnamate (11.0%)	0.4–0.9% (*w*/*w*)	[20,35]
Libya	cis-Chrysanthenol (9.1%), piperitone (30.2%), ethyl cinnamate (3.8%).	0.62% (*w*/*w*)	[23]

Y% refers to the yield of the essential oil.

**Table 2:** Essential oil constituents of *A. judaica* growing in the Northern Qassim region of Saudi Arabia.

RT	Chemical Compounds	Area Mean	*RI^cal^*	*RI^rep^*	*m/z*	Weight g/100 g of the Plant
12.096	(Z)-3-Hexenol	0.50 ± 0.08	845	845		0.0085
12.209	2-Methyl-ethylbutanoate	0.4 ± 0.06	850	853		0.0068
16.403	Sabinene	0.13 ± 0.11	953	954	59.04 (100%), 81.05 (96.37%), 96.07 (83.12%)	0.0022
18.449	α-Phellandrene	1.30 ± 0.16	1000	999	68.04 (100%), 79.03 (42.69%), 93.04 (93.23%)	0.0222
19.795	Limonene	0.72 ± 0.01	1029	1028	85.04 (100%), 55.03 (12.29%), 70.07 (7.28%)	0.0123
20.239	1,8-Cineole	2.56 ± 0.05	1038	1040	69.04 (100%), 110.08 (70.37%), 95.06 (46.98%)	0.0437
21.359	γ-Terpinene	3.58 ± 0.18	1062	1063	135.05 (100%), 91.03 (20.36%), 107.03 (11.26%)	0.0612
23.548	Linalool	1.21 ± 0.04	1108	1104	91.04 (100%), 92.04 (98.97%), 55.04 (47.04%)	0.0207
23.739	α-Thujone	5.94 ± 0.09	1112	1112	95.06 (100%), 81.04 (68.49%), 109.04 (35.48%)	0.1016
24.24	β-Thujone	3.61 ± 0.03	1123	1124	84.0 (100%), 55.02 (80.71%), 126.05 (47.17%)	0.0617
24.49	α-Campholenal	0.91 ± 0.02	1128		82.04 (100%), 110.06 (91.66%), 95.04 (43.11%)	0.0156
24.639	Terpinene-4-ol	6.42 ± 0.17	1132	1140	70.04 (100%), 83.03 (71.89%), 71.03 (29.17)	0.1098
25.443	Isothujol	0.27 ± 0.47	1149	1145		0.0046
25.651	Camphor	1.92 ± 0.09	1154	1155	68.01 (100%), 81.02 (26.97%), 55.04 (18.33%)	0.0328
26.454	Borneol	0.47 ± 0.01	1170	1170	95.04 (100%), 110.04 (48.36%), 54.06 (25.12%)	0.0080
27.156	p-Cymene-8-ol	0.08 ± 0.14	1186	1185	135.06 (100%), 150.08 (40.42), 91.03 (32.05)	0.0014
29.403	Neral	0.36 ± 0.00	1235	1236	69.02 (100%), 68.01 (17.21%), 83.01 (11.68%)	0.0062
30.034	Linalyl acetate	0.10 ± 0.17	1249	1250	107.06 (100%), 95.04 (34.18), 55.03 (14.79%)	0.0017
30.845	Piperitone	31.99 ± 0.50	1268	1260	82.04 (100%), 110.02 (37.44%), 95.06 (19.20%)	0.5470
31.485	Phellandral	0.38 ± 0.02	1281			0.0065
31.965	p-Cymen-7-ol	0.08 ± 0.13	1292	1290		0.0014
32.146	Thymol	1.81 ± 0.03	1296	1297	135.06 (100%), 107.03 (11.26), 77.01 (10.75%)	0.0309
33.406	Carvacrol	0.10 ± 0.12	1325	1324		0.0017
33.643	Citronellyl acetate	0.89 ± 0.02	1330	1334	107.06 (100%), 91.04 (39.11), 122.08 (15.51)	0.0152
34.649	(*E*)-Methyl cinnamate	0.35 ± 0.02	1354	1355	131.02 (100%), 103.03 (61.07), 162.04 (49.31%)	0.0060
35.894	*Cis*-Ethyl cinnamate	4.02 ± 0.06	1383	1376	131.04 (100%), 103.04 (48.29%), 77.03 (30.40%)	0.0687
36.303	Jasmone	0.71 ± 0.02	1392	1396	91.03 (100%), 95.03 (66.88%), 79.03 (60.65%)	0.0121
36.658	β-Bourbounene	4.06 ± 0.17	1401	1401	111.02 (100%), 137.07 (41.99%), 180.09 (21.39%)	0.0694
37.754	β-Caryophyllene	0.43 ± 0.08	1427	1429	161.12 (100%), 105.04 (57.31%), 93.05 (27.09%)	0.0075
39.75	*Trans*-Ethyl cinnamate	13.67 ± 0.55	1477	1455	131.04 (100%), 103.04 (48.29%), 77.03 (30.40%)	0.2337
40.542	Valencene	3.24 ± 0.09	1497	1497	161.12 (100%), 105.04 (57.31%), 91.04 (53.35%)	0.0554
41.138	*γ*-Cadinene	0.79 ± 0.14	1511	1513	161.11 (100%), 133.07 (30.58%), 120.07 (27.73%)	0.0135
41.968	σ-Cadinene	2.37 ± 0.09	1532	1526	91.04 (100%), 205.11 (86.11%), 77.02 (46.25%)	0.04052476
44.321	Spathulenol	3.33 ± 0.07	1593	1575	91.04 (100%), 93.05 (73.39), 77.02 (46.25%)	0.0569
47.198	β-Eudesmol	0.22 ± 0.19	1671	1672	59.04 (100%), 149.11 (67.04%), 146.14 (33.10%)	0.0038
48.344	a-Caryophylene acetate	1.11 ± 0.31	1702	1696	67.04 (100%), 95.06 (62.38%), 96.07 (41.92%)	0.0190
Total		100				1.71
Monoterpene hydrocarbons		5.74				
Oxygenated monoterpenes		57.20				
Sesquiterpene hydrocarbons		10.88				
Oxygenated sesquiterpenes		4.66				
Phenolics		1.87				
Cinnamic acid derivatives		18.03				

RT, Retention time; *RI^cal^*, Calculated retention index; *RI^rep^*, Reported retention index; *m/z*, mass to charge ratio.

**Table 3:** Preliminary antimicrobial activity of ArJ essential oil.

Microorganisms	Zone of Inhibition (mm)	
	ArJ Essential Oil	Control Drugs
*S. aureus* ATCC 29213	7.7 ± 0.20	14.2 ± 0.20
*S. saprophyticus* ATCC 43867	8.8 ± 0.20	12.8 ± 0.20
*S. pyogenes* (A) ATCC 27736	7.4 ± 0.30	11.7 ± 0.10
*S. pneumoniae* ATCC 49619	7.2 ± 0.17	11.7 ± 0.20
*E. faecalis* ATCC 29212	8.7 ± 0.17	11.9 ± 0.10
*B. cereus* ATCC 10876	12.9 ± 0.10	19.6 ± 0.35
*E. coli* ATCC 25922	6.4 ± 0.10	23.1 ± 0.20
*K. pneumoniae* ATCC 27736	6.2 ± 0.10	21.1 ± 0.10
*S. typhimurium* ATCC 13311	10.0 ± 0.20	16.3 ± 0.30
*S. flexneri* ATCC 12022	6.2 ± 0.10	17.9 ± 0.17
*P. vulgaris* ATCC 6380	8.1 ± 0.17	16.2 ± 0.35
*P. mirabilis* ATCC 29906	7.7 ± 0.20	18.7 ± 0.20
*C. albicans* ATCC 10231	25.2 ± 0.20	25.0 ± 0.20
*A. niger* ATCC 6275	15.0 ± 0.20	13.1 ± 0.35

Note: All results are in mean ± SD. Each test was performed in triplicate. Control drugs = levofloxacin (antibacterial), 5 µg/disc; and clotrimazole (antifungal), 50 µg/disc.

**Table 4:** Results of MIC, MBC, MBIC, and MBEC of ArJ essential oil.

Microorganisms	MIC	MBC	MBIC	MBEC
*S. aureus* ATCC 29213	50	100	50	100
*S. saprophyticus* ATCC 43867	50	100	50	100
*S. pyogenes* (A) ATCC 27736	100	>100	100	200
*S. pneumoniae* ATCC 49619	100	>100	100	200
*E. faecalis* ATCC 29212	100	>100	100	200
*B. cereus* ATCC 10876	6.25	12.5	6.25	12.5
*E. coli* ATCC 25922	50	100	50	100
*K. pneumoniae* ATCC 27736	25	50	25	50
*S. typhimurium* ATCC 13311	12.5	25	12.5	25
*S. flexneri* ATCC 12022	12.5	25	12.5	25
*P. vulgaris* ATCC 6380	25	50	25	50
*P. mirabilis* ATCC 29906	100	>100	100	200
*C. albicans* ATCC 10231	6.25	12.5	NT	NT
*A. niger* ATCC 6275	3.125	6.25	NT	NT

Note: All the results are in µL/mL. NT = Not tested. MIC = Minimum Inhibitory Concentration, MBC = Minimum Biocidal Concentration, MBIC = Minimum Biofilm Inhibitory Concentration, MBEC = Minimum Biofilm Eradication Concentration. All the results are in µL/mL.

**Table 5:** Effect of *Artemisia judaica* ointment on antioxidant and oxidant levels in skin burn rat model.

Groups	CAT	SOD	LP
	ng/g		
I. Intact control	1.35 ± 0.05 ^A,B^	0.04 ± 0.00 ^A^	836.9 ± 37.75 ^A^
II. Negative control (skin burn without treatment)	1.11 ± 0.06 ^A^	0.04 ± 0.01 ^A^	1214 ± 51.46 ^B^
III. Silver sulfadiazine	1.79 ± 0.204 ^B^	0.19 ± 0.06 ^A,B^	1197 ± 30.30 ^B^
IV. *Artemisia judaica*	1.82 ± 0.17 ^B^	0.37 ± 0.13 ^B^	1291 ± 18.85 ^B^

Values are denoted as means ± SEM (Supplementary file, Tables S1–S3). Statistical significance was performed using one-way ANOVA, followed by a post hoc test on GraphPad Prism 8.0.2. CAT = catalase, LP = lipid peroxide, SOD = Superoxide dismutase. The mean values that do not share a superscript letter (A,B) in the respective columns of superoxide dismutase (SOD), catalase (CAT), and lipid peroxide (LP) are significantly different (*p* < 0.05) using Tukey’s multi-group comparisons.

**Table 6:** Effect of *Artemisia judaica* ointment on inflammatory and pro-angiogenic markers in skin burn rat model.

Groups	IL-1b	IL-6	TNF-α	TGF-b1	IL-10
	ng/g				
I. Intact control	20.77 ±1.95 ^A^	806.1 ±10.20 ^A^	9.57 ±0.55 ^A^	4.19 ±0.24 ^A^	3.54 ±0.19 ^A^
II. Negative control (skin burn without treatment)	19.37 ± 2.33 ^A^	776.2 ±32.77 ^A^	15.54 ± 0.92 ^B^	3.87 ± 0.09 ^A^	2.99 ± 0.25 ^A^
III. Sulfadiazine	23.55± 0.88 ^A^	789.4 ±18.02 ^A^	12.85± 0.26 ^C^	19.28± 0.30 ^B^	12.68± 0.15 ^B^
IV. *Artemisia judaica*	19.55 ± 1.34 ^A^	869.2 ±51.91 ^A^	11.96 ± 0.34 ^C^	19.18 ± 0.33 ^B^	13.39 ± 0.35 ^B^

Values are denoted as means ± SEM (Supplementary file, Tables S4–S8). Statistical significance was performed using one-way ANOVA, followed by a post hoc test on GraphPad Prism 8.0.2. The mean values that do not share a superscript letter (A–C) in the respective columns of interleukin-1 (IL-1b), IL-6, IL-10, transforming growth factor beta 1 (TGF-b1), and tumor necrosis factor α (TNF-α) are significantly different (*p* < 0.05) using Tukey’s multi-group comparisons.

## Data Availability

Data are available in the manuscript and supplementary file.

## Supplementary Materials

The following supporting information can be downloaded at: https://www.mdpi.com/article/10.3390/antiox11020332/s1, Figure S1: Gas Chromatography (GC), chromatograms of the ArJ essential oil samples; Figure S2: Wound area measurement; Figure S3: Microscopic analysis of skin wound after treatment with SS and ArJ and compared with negative control; Tables S1–S8: The original data of SOD, CAT, LP, IL-1b, IL-6, IL-10, TNF-α, and TGF-b1, respectively.
