# Supplementary material for: Bio-Evaluation of the Wound Healing Activity of Artemisia judaica L. as Part of the Plant’s Use in Traditional Medicine; Phytochemical, Antioxidant, Anti-Inflammatory, and Antibiofilm Properties of the Plant’s Essential Oils
Source: Antioxidants (Basel). 2022 Feb 8;11(2):332. doi: 10.3390/antiox11020332 (PMC8868479; doi:10.3390/antiox11020332)
Supplement: Supplementary file 1 [file antioxidants-11-00332-s001.zip › antioxidants-1570485-supplementary.pdf]

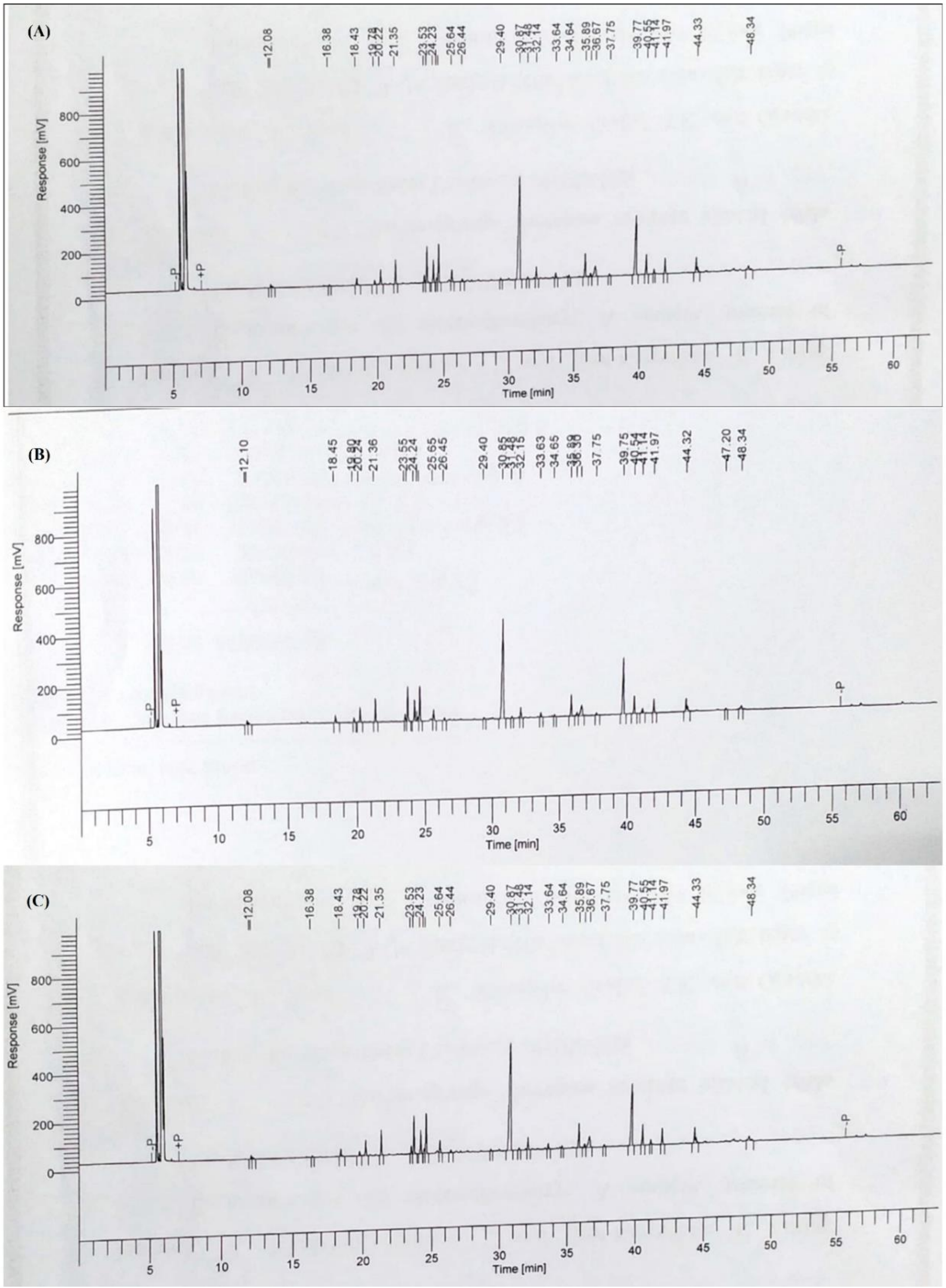

Figure S1: Gas Chromatography (GC), chromatograms for the (A) first ArJ essential oil sample, (B) second ArJ essential oil sample, and (C) third ArJ essential oil sample. The three essential oil samples were obtained from different distillation experiments and injected separately to the GC instrument to calculate the percentages of the volatile oil constituents' ± SD.

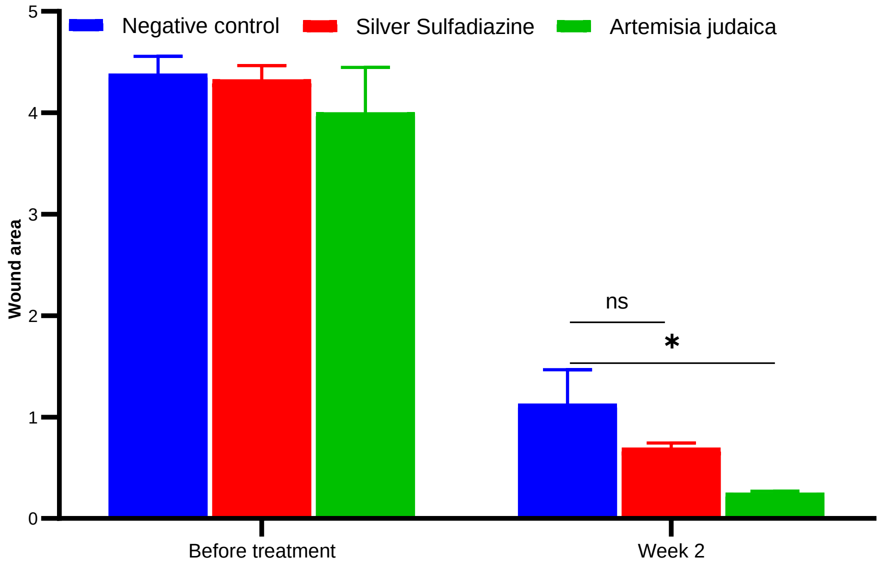

Figure S2 Wound area measurement. * Values are denoted as mean ± SEM. Statistical significance was performed using two-way ANOVA, followed by a posthoc test on GraphPad Prism 8.0.2. ns: non-significant, * significantly different (*P*<0.05) using Tukey's multi-group comparisons.

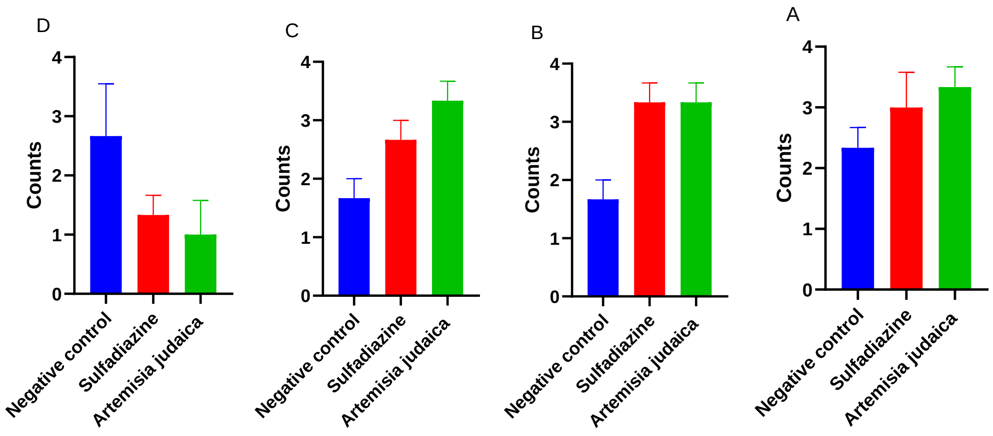

Figure S3. Microscopic analysis of skin wound after treatment with SS and ArJ and compared with negative control for amount of fibroblast (A), collagen (B), neovascularization (C) and inflammation (D). Data was scored from 0-4 where 0, 1, 2, 3, and 4 represented normal, low, moderate, high, and very high, respectively.

	I. Intact control	II. Negative control (skin burn without treatment)	III. Silver Sulfadiazine	IV. Artemisia judaica
	0.050213	0.103255	0.038773	0.046053
	0.050435	0.038773	0.038565	0.043973
	0.04084	0.038149	0.236173	0.567
	0.04129	0.041061	0.32	0.612
	0.031	0.016181	0.35	0.595
**Average**	**0.043**	**0.047**	**0.197**	**0.373**
**median**	**0.041**	**0.039**	**0.236**	**0.567**
**S.D**	**0.008**	**0.033**	**0.150**	**0.300**
**n**	**5.000**	**5.000**	**5.000**	**5.000**
**S.E**	**0.004**	**0.015**	**0.067**	**0.134**

Table S1: Original data of SOD

	I. Intact control	II. Negative control (skin burn without treatment)	III. Silver Sulfadiazine	IV. Artemisia judaica
	1.261	1.265	1.301	2.032
	1.495	1.214	1.863	1.625
	1.321	0.944	2.321	1.247
	1.234	1.16	2.12	2.213
	1.461	1.011	1.36	1.984
**Average**	**1.354**	**1.119**	**1.793**	**1.820**
**median**	**1.321**	**1.160**	**1.863**	**1.984**
**S.D**	**0.118**	**0.136**	**0.453**	**0.385**
**n**	**5.000**	**5.000**	**5.000**	**5.000**
**S.E**	**0.053**	**0.061**	**0.203**	**0.172**

Table S2: Original data of CAT

	I. Intact control	II. Negative control (skin burn without treatment)	III. Silver Sulfadiazine	IV. Artemisia judaica
	937.6	1301	1213.6	1321.1
	888.1	1121	1211.6	1221.1
	724	1186	1121.1	1324.1
	784	1098	1145	1284
	851	1364	1294	1304
**Average**	**836.940**	**1214.000**	**1197.060**	**1290.860**
**Media**	**851.000**	**1186.000**	**1211.600**	**1304.000**

n				
S.D	84.418	115.063	67.754	42.147
n	5.000	5.000	5.000	5.000
S.E	37.753	51.458	30.301	18.849

Table S3: Original data of LP

	I. Intact control	II. Negative control (skin burn without treatment)	III. Silver Sulfadiazine	IV. Artemisia judaica
	21	13.27	26.27	19.77
	23	15.57	22.97	23.57
	18	19	20.97	15.77
	26.57	26	24.56	17.65
	15.27	23	23	21
Average	20.768	19.368	23.554	19.552
median	21.000	19.000	23.000	19.770
S.D	4.376	5.218	1.982	3.009
n	5.000	5.000	5.000	5.000
S.E	1.957	2.334	0.886	1.346

Table S4: Original data of IL-1b

	I. Intact control	II. Negative control (skin burn without treatment)	III. Silver Sulfadiazine	IV. Artemisia judaica
	844.22	735.33	842	862
	795.33	753.11	766.44	999.78
	793.11	859.78	737.56	704.22
	810	688.67	789	956
	788	844.22	812	824
Average	806.132	776.222	789.400	869.200
median	795.330	753.110	789.000	862.000
S.D	22.807	73.276	40.287	116.080
n	5.000	5.000	5.000	5.000
S.E	10.200	32.770	18.017	51.913

Table S5: Original data of IL-6

	I. Intact control	II. Negative control (skin burn without treatment)	III. Silver Sulfadiazine	IV. Artemisia judaica
	3.14	3.21	12.59	14.14
	3.78	2.81	12.7	12.92

	3.86	3.87	12.7	13.59
	3.94	2.63	13.21	14.06
	3.01	2.47	12.21	12.26
**Average**	**3.546**	**2.998**	**12.682**	**13.394**
**median**	**3.780**	**2.810**	**12.700**	**13.590**
**S.D**	**0.436**	**0.560**	**0.357**	**0.798**
**n**	**5.000**	**5.000**	**5.000**	**5.000**
**S.E**	**0.195**	**0.250**	**0.160**	**0.357**

Table S6: Original data of IL-10

	I. Intact control	II. Negative control (skin burn without treatment)	III. Silver Sulfadiazine	IV. Artemisia judaica
	11.05	15.45	13.13	11.47
	8.47	16.78	12.13	12.55
	9.56	15.26	12.47	11.13
	10.56	12.35	13.64	12.99
	8.21	17.86	12.87	11.68
**Average**	**9.570**	**15.540**	**12.848**	**11.964**
**median**	**9.560**	**15.450**	**12.870**	**11.680**
**S.D**	**1.248**	**2.073**	**0.584**	**0.777**
**n**	**5.000**	**5.000**	**5.000**	**5.000**
**S.E**	**0.558**	**0.927**	**0.261**	**0.348**

Table S7: Original data of TNF-α

	I. Intact control	II. Negative control (skin burn without treatment)	III. Silver Sulfadiazine	IV. Artemisia judaica
	4.2	3.71	20.29	19.79
	3.64	3.86	19.41	19.95
	5.1	3.66	19.33	19.33
	4.13	4.2	18.45	18.62
	3.9	3.93	18.91	18.21
**Average**	**4.194**	**3.872**	**19.278**	**19.180**
**median**	**4.130**	**3.860**	**19.330**	**19.330**
**S.D**	**0.552**	**0.213**	**0.683**	**0.749**
**n**	**5.000**	**5.000**	**5.000**	**5.000**
**S.E**	**0.247**	**0.095**	**0.305**	**0.335**

Table S8: Original data of TGF-b1
